# Adapting the Primary Care Assessment Tool for sub-Saharan Africa: a validation study

**DOI:** 10.3399/BJGPO.2024.0084

**Published:** 2025-01-29

**Authors:** Robert Mash, Kefilath Bello, Innocent K Besigye, Anna Galle

**Affiliations:** 1 Division of Family Medicine and Primary Care, Stellenbosch University, Cape Town, South Africa; 2 Centre de Recherche en Reproduction Humaine et en Démographie, Cotonou, Benin; 3 Department of Family Medicine, Makerere University, Kampala, Uganda; 4 Department of Public Health and Primary Care, WHO Collaborating Centre on Family Medicine and Primary Health Care, Ghent University, Ghent, Belgium

**Keywords:** primary health care, questionnaire design, Delphi technique, quality assurance, Africa South of the Sahara

## Abstract

**Background:**

The World Health Organization’s (WHO) measurement framework for primary health care includes the core functions of primary care: first-contact access, comprehensiveness, coordination, continuity, and person-centredness. The Primary Care Assessment Tool (PCAT), originally developed in the USA, was adapted for use by four African countries, and can measure the core functions of primary care.

**Aim:**

To face and content validate a PCAT for sub-Saharan Africa that measures the core functions of primary care.

**Design & setting:**

Nineteen countries within the Primary Care and Family Medicine (PRIMAFAMED) network for sub-Saharan Africa participated in a validation study.

**Method:**

Two stages included a PRIMAFAMED workshop to assess face validity and a Delphi study to reach consensus on content validity among an expert panel as well as key stakeholders.

**Results:**

Thirteen countries participated in the workshop and suggested rephrasing 39 items, deleting six and adding four new items. Nineteen countries participated in the Delphi study and all 20 panel members reached consensus (>70%) on including the items as written. Seven experts and stakeholders reviewed the PCAT and suggested rephrasing 23 items, deleting one and adding one. The final PCAT for sub-Saharan Africa (SSA-PCAT) consists of 85 items that measure affiliation with the primary care facility, first-contact access and utilisation, comprehensiveness, continuity, coordination, and person-centredness, as well as health, demographic and socioeconomic status.

**Conclusion:**

The SSA-PCAT will now be piloted in Benin, Uganda, and South Africa. Further psychometric evaluation will be possible followed by more widespread use by researchers, district managers, and policymakers in the region.

## How this fits in

The World Health Organization is looking for tools to measure the core functions of primary care, as per its measurement framework. The Primary Care Assessment Tool (PCAT) had been previously adapted for use in four African countries and had the potential to measure the core functions. This study adapted and validated the tool for use throughout sub-Saharan Africa (SSA) and to specifically measure the core functions. This tool can be used to compare primary care performance across settings and countries and improve overall primary care performance in the region.

## Introduction

In 2018, the Astana Declaration confirmed a global commitment to strengthen primary health care (PHC).^1^ Following this declaration, the World Health Organization (WHO) published an operational plan and a measurement framework.^2,3^ The operational plan defined PHC as having the following three key components: integrated primary care services with key public health functions; empowered communities; and multisectoral policy and action. The measurement framework followed a logic model with health system structures and inputs supporting service delivery activities and outputs, which led to health system outcomes and impact. Service delivery outputs were divided into access and utilisation, as well as quality of care.

In the WHO measurement framework, the quality of primary care included the core functions, effectiveness, efficiency, and safety.^3^ Five core functions were defined and included first-contact access, continuity, coordination, comprehensiveness, and person-centredness. These are cross-cutting functions that define high-quality primary care as opposed to disease or programmatic oriented indicators. Most health systems monitor the key health system inputs such as infrastructure, workforce, and supply of medicines, as well as disease-oriented outcomes such as viral suppression in people with HIV or tuberculosis (TB) treatment success rates.^4,5^ Although the core functions are important to measure, there are few tools available. The Primary Health Care Performance Initiative (PHCPI) struggled to find routine indicators that could measure the core functions.^6^ The WHO suggests that they are measured by exit interviews with patients.^3^

In sub-Saharan Africa (SSA) several countries have adapted and validated the Primary Care Assessment Tool (PCAT). This was originally developed in the USA in the 1990s and designed to measure four of the core functions (excluding person-centredness) as well as other constructs such as community orientation, cultural competence, and family orientation.^7^ The original PCAT was found to be a reliable and valid tool.^8^ The South African Primary Care Assessment Tool (ZA-PCAT) was adapted with the addition of a domain to measure the PHC team and used within the public sector.^9^ The Kenyan Primary Care Assessment Tool (KE-PCAT) was validated and used within the private sector.^10^ When the Ugandan Primary Care Assessment Tool (UG-PCAT) was adapted the research team added the construct of person-centredness.^11^ In Malawi, the research team took a different approach and performed exploratory factor analysis on the items.^12^ This resulted in different constructs and a reduced set of items in the Malawi Primary Care Assessment Tool (MW-PCAT). The adapted versions in South Africa, Kenya, and Uganda stayed close to the original design.

The researchers in these different SSA countries were also members of the PRIMAFAMED (Primary Care and Family Medicine) network,^13^ and realised that the different versions of the PCAT could potentially be combined into a regional version. With the advent of the new WHO measurement framework, they also realised that PCAT could be streamlined to just measure the core functions of primary care. This article describes the process of face and content validation of a sub-Saharan version of the PCAT, measuring the core functions of primary care in the region.

## Method

### Study design

This study had a two-step procedure: first, assessing face validity by a face-to-face workshop with experts; and second, conducting a more in-depth content validity procedure by means of a Delphi study.

### Setting

The research project was embedded in a broader initiative, aiming to develop WHO collaborating centres for PHC in SSA. The project was initiated by the authors as part of this initiative in collaboration with the PRIMAFAMED network.^13^ PRIMAFAMED is a network of 40 departments of family medicine and primary care from 25 countries in SSA. An annual meeting brings together at least one person from each country in the network, together with international partners. The PCAT research project was endorsed by both PRIMAFAMED and the WHO African Region (WHO AFRO).

### Instrument

The five core functions of PHC were defined as follows:^3^

- First-contact access: how easy is it for users to access primary care as the first place that they go when looking for assistance with a health issue.
- Continuity: informational continuity focuses on whether an up-to-date medical record is available when people attend primary care; relational continuity focuses on whether they develop a relationship of trust with and are known by their primary care provider
- Coordination: parallel coordination focuses on coordination between teams within the PHC level of care, sequential coordination focuses on coordination between teams in different levels of care.
- Comprehensiveness: focuses on whether primary care covers the lifespan, the burden of disease, and includes health promotion, disease prevention, treatment, palliation, and rehabilitation.
- Person-centredness: focuses on whether care incorporates both the biomedical and users’ perspectives on the illness, and whether care focuses on the person and not just the disease.

The PCAT also contained items to measure affiliation to primary care providers or facilities, self-reported health status, socioeconomic and demographic characteristics.

### Data collection procedures

#### Step 1: Face validity of a draft regional PCAT

Before the PRIMAFAMED workshop, the first author (RM) reviewed three of the existing versions of the PCAT instrument (ZA-PCAT, KE-PCAT, and UG-PCAT). Notes were made on any variations found between the different versions. The document was made available to all participants at the workshop.

The 4-hour workshop started with an introduction to PCAT, the goals and process of the workshop. This was followed by presentations on the local adaptation and validation processes for PCAT in South Africa, Kenya, and Uganda.

A 'world cafe' process followed the presentations, consisting of five stations, one for each of the core functions. Each station had a facilitator and the participants spent 25 minutes at each station. When participants attended the station, the facilitator gave a brief summary of the feedback from previous groups and then discussed the section of the PCAT with the new group. Facilitators recorded all feedback by pen and paper. After the workshop each facilitator provided a summary of the group’s recommendations to the first author (RM). The research team resolved any contradictions or unclear feedback on discussion.

#### Step 2: Content validity by an online Delphi process to reach consensus on the contents of the regional PCAT

An expert panel was invited to participate in an online Delphi process with one participant from each country represented in the PRIMAFAMED network. These were academic family physicians with an understanding of both PHC in their own countries and primary care research. Twenty people participated in the panel from 19 countries (Ghana, Ethiopia, Nigeria, Zimbabwe, Zambia, Uganda, Lesotho, Tanzania, Democratic Republic of Congo, Benin, South Africa, Malawi, Somaliland, Rwanda, Kenya, Botswana, Eswatini, Namibia, Mozambique).

The first author used the recommendations of the PRIMAFAMED workshop to design a draft regional PCAT in collaboration with the other researchers. A questionnaire was developed in REDCap and panel members were individually invited through an email generated by REDCap, which had a unique link to the questionnaire. Panel members could respond to each item with a recommendation of 'keep this item as it is', 'keep this item, but rephrase' or 'delete this item as not relevant'. For each of the sections in the questionnaire, the responders had the option to give qualitative feedback. They could either suggest how to rephrase the item or suggest additional items. The researchers agreed that consensus would be reached when>70% agreed on a designation for an item.

Up to three rounds of the Delphi process were planned. In each round the items on which consensus was reached would be removed and only new items or items on which there was no consensus presented again. The panel would also receive feedback on the proportion of votes for each option and the qualitative feedback. If, after three rounds, there were still items with no consensus then a virtual meeting of the panel would discuss the remaining items and reach agreement.

As the Delphi panel consisted of family physicians within the PRIMAFAMED network, another round of feedback from additional stakeholders was planned as a last step of the Delphi process. The three African researchers were from East, Southern, and West Africa (Uganda, South Africa, and Benin) and decided to identify 2–3 additional stakeholders in their own countries. They looked for experts and policymakers in public health and PHC measurement from the WHO country office and from the Department of Health.

Participants were asked to give feedback on the version of the regional PCAT derived from the first round of the Delphi process. They were asked to comment on the relevance of items, and the need to rephrase items or any missing items. All the feedback from the participants was integrated by the research team and the Delphi process was concluded with consensus on a regional PCAT.

## Results

Representatives from 13 countries participated in the PRIMAFAMED workshop (Benin, Belgium, Republic of Ireland, Uganda, Tanzania, South Africa, Ethiopia, Kenya, Zambia, Lesotho, Namibia, Nigeria, Botswana). Overall, participants recommended rephrasing 39 items, deleting six and adding four new items.

Twenty representatives from 19 countries participated in the Delphi study. In the first round, all items were approved as formulated by>70% of the panel. Qualitative feedback from the participants was still reviewed by the research team. As consensus was obtained in round 1, there was no need to proceed to further rounds.

In the final consultation with seven additional stakeholders, we considered feedback from three country-level WHO offices, three departments of health, and one additional PHC researcher in Benin. They made 23 suggestions on rephrasing items, suggested one deletion and one addition on financial barriers to access.

Feedback was integrated by the research team and a final PCAT version was drafted. The final items included in the SSA-PCAT for each of the core functions are presented in Tables 1 [Table table2] [Table table3] [Table table4]-5.

**Table 1.** Items to measure access and utilisation

**Utilisation**	
B1	Is this facility the first place you go to when you need a regular check-up?
B2	Is this facility the first place you go when you have a new health problem?
B3	Do you need a letter or appointment from this facility before you can get services from a specialist at the hospital?
**Access**	
C1	Is your facility open on weekends?
C2	Is your facility open in the evening for at least some weekdays?
C3	When your facility is closed, is there a phone number you can call when you get sick?
C4	When your facility is closed and you get sick, would someone from there visit you at home?
C5	Do the fees make it difficult for you to obtain care at this facility?

**Table 2. table2:** Items to measure comprehensiveness

The following is a list of services that you or your family might need at some time. For each one, please indicate whether it is available at your facility	
D1	Vaccinations/immunisations/injections to prevent diseases in children, for example, polio
D2	Vaccinations/immunisations/injections to prevent diseases in adults, for example COVID-19, flu
D3	Dental check-up; checking and cleaning your teeth
D4	Family planning or birth control methods, for example, pills, injections, intrauterine device
D5	Screening for cervical cancer
D6	Counselling on safe sex, for example, to prevent HIV or sexually transmitted diseases
D7	Counselling to stop smoking
D8	Advice about healthy diet
D9	Monitoring growth and development in a child
D10	Counselling for social problems, for example, family conflicts, social grants
D11	Advice about appropriate physical activity, for example, walking, gardening, housework, sport
D12	Alcohol or drug abuse counselling or treatment
D13	Counselling or treatment for mental health problems
D14	TB testing
D15	Stitching up a bad cut
D16	Counselling and testing for HIV/AIDS
D17	Checking your hearing
D18	Checking your eye sight
D19	Immobilisation of fractures, for example, splint, plaster cast
D20	Checking your blood pressure
D21	Checking and discussing medications you are taking
D22	Care for pregnant women, that is, antenatal care
D23	Treatment for ingrown toenails, that is, removing part of the toenail
D24	Help someone obtain assistive devices for disability, for example, wheelchair, walking stick
D25	Provide adequate pain relief for someone with a terminal or life-threatening illness, for example, cancer
D26	Provide guidance when someone in your family cannot make decisions about his/her care, for example, very old (senile) or severe mental illness
D27	Support older adults when there are changes in mental or physical abilities, for example, when too frail, memory loss
D28	Organise home-based care, for example, a visit from a community health worker
D29	Obtain medication for high blood pressure or diabetes

TB = tuberculosis

**Table 3. table3:** Items to measure continuity of care

E1	When you come to this facility are you taken care of by the same health provider each time?
E2	If you have a question, can you easily come back and talk to the health provider who treated you before?
E3	Does your health provider know you very well as a person?
E4	Does your health provider know what is most important to you in terms of your health?
E5	When you go to your facility, is your medical file always available?
E6	Does your health provider have access to your complete medical history?
E7	Does your health provider know about all the medications you are taking? (for example, got elsewhere, traditional medicines)
E8	If you had the choice, would you change your facility to somewhere else?

**Table 4. table4:** Items to measure person-centredness

When you visit this facility, do health professionals usually …	
F1	Greet you in an acceptable way?
F2	Allow you to fully express your thoughts concerning your health problems?
F3	Listen carefully to what you have to say?
F4	Check to be sure they have understood everything?
F5	Give you as much information as you want?
F6	Check to see if the treatment plan is acceptable to you?
F7	Spend enough time with you?
F8	Involve you in decisions about your health as much as you want?
F9	Allow or invite you to ask questions or raise concerns?
F10	Overall, are you satisfied with your visits to the healthcare workers at this facility?

**Table 5.** Items to measure coordination

G1	Are the results of investigations (for example, blood tests, sputum tests, X-rays) explained to you?
G2	Do your healthcare providers in this facility work well together to come up with one coordinated plan for you (for example, between different services in the same facility)?
G3	Do your healthcare providers inside the facility coordinate well with those working outside the facility, in the community (for example, nurses in the facility with community health workers outside)?
**G5–G9 only answered if the person was referred to a specialist or hospital**	
G5	Did your healthcare provider at this facility send you to the specialist or hospital?
G6	Did someone at this facility make the appointment for your first visit to the specialist or hospital?
G7	Did this facility give you a letter for the specialist or hospital about the reason for the visit?
G8	Did this facility know what the outcome of the visit was, did they receive any information back from the specialist or hospital?
G9	After you went to the specialist or hospital, did someone at this facility talk with you about what happened at the hospital visit?

## Discussion

### Summary

This process of face and content validation resulted in a novel version of the PCAT for SSA with consensus from 20 representatives from 19 countries. This included English, French, and Portuguese-speaking countries as well as countries from West, East, Central, and Southern Africa. The SSA-PCAT version only included domains to measure the five core functions of primary care. Other domains that were included in the original and earlier African versions were excluded to align the tool with the WHO measurement framework and increase the utility of the tool.

The final SSA-PCAT has 85 items, and although this is more concise than previous African versions (UG-PCAT had 111 items), it is longer than the original USA short version that had 62 items. Comparison with the original validated US version of the PCAT provides a perspective on how Africa has adapted the tool (Supplementary Table 1)^7^ The SSA-PCAT did not attempt to separate domains into structural and process items as in the original USA tool (for example, comprehensiveness available versus provided). The domain on coordination (information systems) was incorporated into continuity of care as it focuses on what we now call informational continuity.^14^ Additional items were added to coordination to measure both parallel and sequential coordination of care, as per current thinking on these concepts.^14^ Person-centredness can be a difficult construct to measure,^15^ but the new items were taken from a tool that had been previously validated in Kenya.^16^ The SSA-PCAT added 21 new items to the domain on comprehensiveness.

### Strengths and limitations

The process of face and content validation included a broad range of country representatives and stakeholders and a variety of methods to ensure consensus. Not all countries in SSA participated and it is possible that some settings would disagree with some of the items. The process did not include any community members or patient representatives and this voice might have added a useful additional view. The process did not formally test the psychometrics of the tool, since this has been done previously.^8^ Nevertheless, as this is a new version of the PCAT, it would be helpful to assess the internal reliability during the pilot phase and conduct a confirmatory factor analysis. Such factor analysis might enable a further reduction in the number of items, particularly for comprehensiveness. Furthermore, it needs to be recognised that validation is an ongoing process and additional studies might complement our work.

### Comparison with existing literature

Most health information systems in SSA do not measure the core functions of primary care.^17^ For example, in South Africa the Ideal Clinic norms and standards are measured annually and include many aspects of the WHO’s health system inputs and service delivery activities and outputs.^18^ However, they do not include measures of the core functions of primary care. The SSA-PCAT therefore has the potential to complement existing health information systems. Measuring the core functions also helps managers and primary care providers to move away from a disease-oriented and programmatic view of performance towards a more cross-cutting, holistic, and functional approach. District-level managers may not be that familiar with the concepts included in the core functions (personal communication, IB from his PCAT work in Uganda) and the SSA-PCAT might set the scene for evaluating, improving, and rethinking the organisation of PHC in the region.

Providing a tool to measure the core functions is an essential part of the process of performance management and improvement.^3^ It is important that the data collected are reliable, analysed, and used to give feedback to district-level management teams in an easy-to-use format. Management teams will need to be supported to make sense of the findings, identify the root causes of poor functionality and to design, develop, and implement interventions to improve performance. In that aspect, introducing the SSA-PCAT instrument to measure performance of the PHC system will need to be accompanied with mentoring and appropriate guidance.

It also needs to be recognised that healthcare workers have limited time, interest, and capability to reflect on routinely collected data.^19^ Performance management may be seen as a *'top-down hierarchical activity, driven by fear of authority, and supported by control of financial and human resources'*.^19^ There is often a lack of supportive supervision, insufficient data officers, little information technology, and low capability in health informatics.^19^ Providing a useful tool to measure the core functions of primary care is therefore only one piece of the puzzle in improving quality of care.

### Implications for research and practice

The SSA-PCAT meets the requirements of the WHO measurement framework for a tool that measures the core functions of primary care in exit interviews with users.^3^ The SSA-PCAT should be adopted by WHO AFRO as part of the toolkit for countries to use. The tool can be used in a variety of ways. Researchers may use the SSA-PCAT to measure the core functions as an outcome in experimental or observational studies. Service delivery managers and clinicians may use the SSA-PCAT to identify gaps in performance and guide quality improvement. Health systems can include the SSA-PCAT as a periodically collected measure within the national dataset and required data to monitor performance. For example, in South Africa this would complement the annual audit of PHC facilities (Ideal Clinic project), which focuses more on health system inputs and outputs, and does not measure the core functions.^20^ Its value lies in its ability to enable performance management and improvement processes in PHC as these processes are often weak in SSA.^19^

The SSA-PCAT will be piloted in three districts, one in Benin, Uganda, and South Africa, by the three African researchers leading this study. Afterwards we intend to disseminate its use across 10 countries in SSA, facilitated by the PRIMAFAMED network. This research will further build the validity and utility of the tool.

In conclusion, this study has validated a sub-Saharan version of the PCAT that can be used to measure the core functions of primary care, as per the new WHO measurement framework. The SSA-PCAT will be further piloted in Benin, Uganda, and South Africa. Afterwards, the SSA-PCAT will be made widely available for researchers, district management teams, and health information systems across the region.

## Data

The dataset relied on in this article is available from the corresponding author on reasonable request.
